# Purchasing health services under the Egypt's new Universal Health Insurance law: What are the implications for universal health coverage?

**DOI:** 10.1002/hpm.3354

**Published:** 2021-11-01

**Authors:** Ahmed Yehia Khalifa, Jean Yacoub Jabbour, Awad Mataria, Magdy Bakr, Mai Farid, Inke Mathauer

**Affiliations:** ^1^ World Health Organization Representative Office Cairo Egypt; ^2^ Eastern Mediterranean Regional Office World Health Organization Cairo Egypt; ^3^ Ministry of Finance Egypt; ^4^ World Health Organization Headquarter Geneva Switzerland

**Keywords:** Egypt, health financing, health insurance, health service purchasing, universal health coverage

## Abstract

**Background:**

Egypt's Universal Health Insurance (UHI) Law of 2018 implies major transformation to the health financing system. This commentary provides an assessment of the purchasing arrangements as stipulated by the UHI Law and Bylaw, their implications and contribution to progress towards universal health coverage (UHC). The purpose of this assessment is to inform the multi‐year implementation process of the Law and propose options for progress towards UHC.

**Methods:**

Guided by an analytical framework on purchasing, the qualitative analysis was based on the review of the legal provisions and structured discussions with key stakeholders.

**Results:**

The Law foresees important changes, such as a purchaser‐provider split, stricter referral rules and regulated cost‐sharing. However, several purchasing aspects were not sufficiently specified in the legal provisions, for example benefit design and provider payment methods. It remains unclear for decision‐makers how to proceed, hindering the Law's effective implementation. There are also concerns about the mixed provider payment system creating incoherent provider incentives.

**Conclusion:**

In view of the remaining legal unclarities on purchasing, progress towards UHC is restrained. Benefits design and the provider payment system should be further specified with a clearer governance structure around the purchasing decision‐making processes. Additional technical options for strategic purchasing are suggested.

## Key points

- Egypt's new Universal Health Insurance (UHI) Law sets the potential for progress towards Universal Health Coverage and implies major transformation to the health financing functions, that is revenue raising, pooling, and purchasing of health services
- While there are important institutional changes in the purchasing arrangements to shift towards more strategic purchasing, the persistence of a mixed and non‐aligned provider payment system with incoherent incentives may lead providers to engage in resource‐shifting and cream skimming of patients
- Further specifications, explicit criteria and clear processes are needed to set the benefits and to establish the new payment system
- A key lesson of the Egyptian case relevant for other countries is that a legally binding mandate is critical to establish health financing and purchasing reforms, however, alignment across different elements of the reform is equally critical to ensure that purchasing reforms have a positive impact to improve access to care and financial protection. It is also important to consider the political economy that lies behind ‘technical’ options

## 1 INTRODUCTION

Egypt issued a Universal Health Insurance (UHI) Law in 2018 in order to expand health coverage to its population. With implementation having started in July 2019, the law has an enormous potential to stimulate substantial progress towards Universal Health Coverage (UHC). When fully implemented, over a period of 12–15 years, it is envisaged that all Egyptians will be covered under the UHI scheme, with a benefit package of quality health services and financial protection. This law implies major implications on Egypt's health system and in particular on health financing, that is how resources are raised, pooled and used to purchase health services.

This article focuses on the purchasing function. For progress towards UHC, purchasing needs to be strategic, that is it needs to be based on deliberate and informed decisions on which services to purchase, from whom (which provider types and levels), and how to pay providers.^1^ The key objectives of strategic purchasing are to realize efficiency gains, improve equitable access and financial protection, as well as to increase quality of health services. However, various critical purchasing aspects were not sufficiently specified in Egypt's UHI Law and its Bylaw. As such, it remains unclear for decision makers how to proceed and this may hinder the Law's effective implementation.

This article analyses the purchasing arrangements as stipulated by the UHI Law and Bylaw, their implications and related implementation challenges, and how they affect progress towards UHC objectives, that is, efficiency, equitable access, quality and financial protection. Our assessment aims to inform the ongoing long‐lasting implementation process of the Law and propose options to support a shift towards more strategic purchasing in order to accelerate progress towards UHC.

## 2 METHODS

For this paper, we focused on core purchasing elements, namely benefits design and specification, the conditions of access, the process of setting provider payment methods and their alignment.

Our qualitative analysis was oriented by an analytical framework that serves to assess a country's purchasing arrangements.^2^ The framework provides a set of guiding questions on the various purchasing arrangements and their desirable attributes. Moreover, our assessment was oriented by an analytical guide with key questions that focus on assessing a mixed provider payment system.^3^ Drawing on these frameworks, Table 1 outlines the key elements that were explored.

**TABLE 1:** Key aspects explored to guide the country analysis

Key component of strategic purchasing	Design aspects conducive to UHC
Benefits specification	‐ A well‐defined process with clear criteria on benefits design is in place. ‐ People are made aware of their entitlements. ‐ Benefits are specified with clear decisions on treatment options and medicines; they focus on cost‐effective, primary health care (PHC) services, including prevention and promotion, and on the disease burden of the vulnerable. ‐ A regular revision process to update the benefits is in place.
Cost‐sharing mechanisms	‐ Cost‐sharing mechanisms and referral rules or gatekeeping mechanisms are clearly specified. ‐ Cost‐sharing rates are differentiated for different care levels (e.g., they could be higher for higher level care) and for different income groups (e.g., poorer population groups could be exempted).
Process of setting payment methods and rates	‐ Payment methods are chosen in line with the core objectives for the health system. ‐ A clear process for setting payment rates and monitoring its impact on the providers and population is in place. ‐ Providers have a sufficient level of financial autonomy to respond to incentives created by the payment methods and rates.
Alignment of payment methods	‐ The provider payment system constitutes an aligned mix to create a coherent set of incentives for providers to reduce overprovision and under‐provision and to avoid resource shifting including patient cream skimming and cost shifting.

*Note*: Here we are listing only the aspects that this paper assesses. The country assessment was more comprehensive and hence was based on a much longer list of key aspects and related questions.

*Source*: adapted from^2^, ^3^.

This country analysis partly builds upon an earlier assessment of purchasing and governance arrangements which was undertaken as part of the perennial World Health Organization's (WHO) policy advisory support to the Government of Egypt related to the UHI Law development and implementation.^4^ At first, we construed the UHI Law and Bylaw,^5^, ^6^ followed by discussions coupled with clarification and reflection questions with key stakeholders in health purchasing (see Table 2).

**TABLE 2:** Overview of stakeholders interviewed

Stakeholders (number of interviews)	Further details
Ministry of Health and Population (MoHP) (7)	Interviewees represented the ministry's main sectors and departments including curative care, primary care, infrastructure, the health information center, the MoHP technical office, the pharmacoeconomic unit, and the ‘Programme for the Treatment at the Expense of the State’
Ministry of Finance (3)	Interviewees represented the public treasury, budget sector and Economic Justice units
Current Health Insurance Organization (1)	A former vice chairman
Chamber for private health providers (1)	A private sector representative
Members of the temporary costing committee (10)	The interviewees included independent experts and representatives of health providers
UHI Law drafting committee member (1)	The interviewee represented the UHI Law drafting committee in addition to being independent expert

Moreover, as part of the currently ongoing WHO policy advisory support to the Government and in particular through the participation in the pricing and strategic purchasing committees by one of the authors, the early actual implementation process and further developments in the legal provisions as well as reform efforts relating to health purchasing over a period of 16 months since the law came into power in July 2019 were recorded and evaluated.

## 3 Understanding the context: the old and the new health system and financing architecture

This section provides some context and sketches out the financing architecture before and after the implementation of the UHI Law. Egypt's healthcare system has been challenged by underinvestment and inefficiencies.^7^ Over the past decade, household out‐of‐pocket payments (OOPS) constituted around 62% of current health expenditure, compared to the average of 39% in lower‐middle income (LMICs), while government spending on health as a share of current health expenditure represented a rather stagnant rate of around 29% compared to 43% in LMICs.^8^ Based on latest data of 2012 presented in the 2019 Universal Health Coverage (UHC) Global Monitoring Report, 3.9% of the Egyptian population were faced with severe catastrophic health expenditure (Severe catastrophic expenditure is large out‐of‐pocket expenditure [>25%] in relation to household income or consumption), and 1.07% (Poverty line: at 2011 PPP $3.20‐a‐day) were impoverished due to direct out‐of‐pocket payment.^9^ Table 3 outlines key health indicators while Table 4 presents core health expenditure indicators, revealing the need to increase general government expenditure on health.

**TABLE 3:** Key health indicators

	Egypt	LMIC
Maternal mortality ratio (per 100,000 live births)	33 [26–39]^a^	260^a^
Probability of dying under five (per 1000 live births)	22^b^	49
Life expectancy male/female	68/73^c^	66/70^b^
DTP3 Immunization coverage	94%^b^	82%^b^
Number of doctors per 1000 population	0.79^b^	0.7^d^

Abbreviation: LMIC, Lower middle‐income countries.

^a^ Estimates for 2015.

^b^ estimates for 2017.

^c^ estimates for 2016.

^d^ estimates for 2013.

*Source*: ^10^, ^11^.

**TABLE 4:** Health expenditure indicators, 2018

	Egypt	LMIC
Current Health Expenditure (CHE) as a % of Gross Domestic Product (GDP)	4.9	5.1
Domestic General Government Health Expenditure (GGHE‐D) as % Current Health Expenditure (CHE)	28.7	42.5
Out‐of‐pocket (OOP) as % of Current Health Expenditure (CHE)	62.2	39.0
Domestic General Government Health Expenditure (GGHE‐D) as % General Government Expenditure (GGE)	5.4	7.3
Domestic General Government Health Expenditure (GGHE‐D) as % Gross Domestic Product (GDP)	1.4	2.3
General Government Expenditure (GGE) as% of Gross Domestic Product (GDP)	30.1	30.4

Abbreviation: LMIC, Lower middle‐income countries.

*Source*: Latest health account estimates based on reference World Health Organization.^8^

**FIGURE 1:**
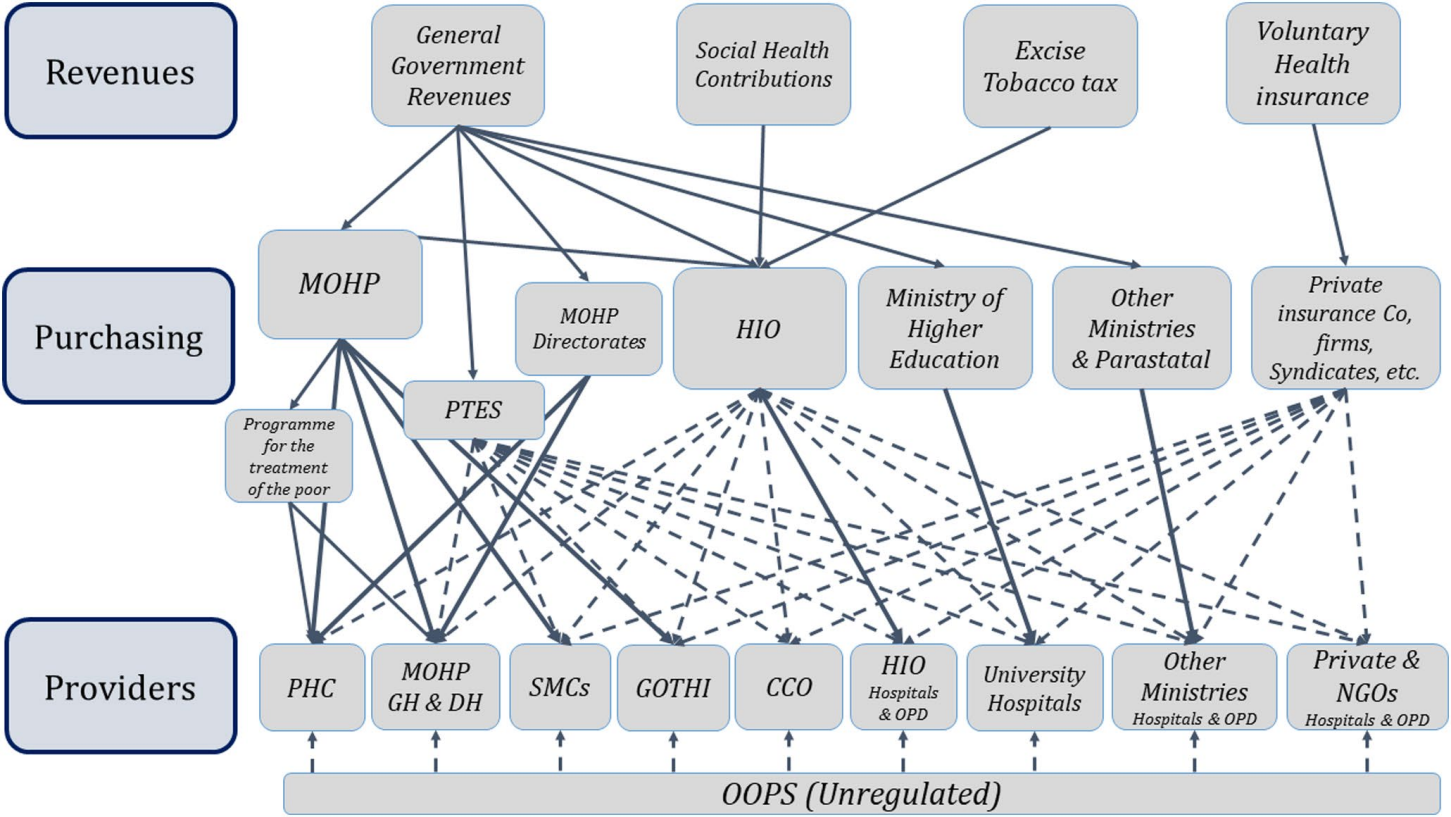
Current health financing system architecture and funding flows. For demonstration purposes. HIO was represented in one box (pool) although it is comprised of several pools. MOHP, Ministry of Health and Population; HIO, Health Insurance Organization; PTES, Programme for the Treatment at the Expense of the State; PHC, Primary Health Care; GH, General Hospitals; DH, District Hospitals; SMCs, Specialized Medical Centers; GOTHI, General Organization for Teaching Hospitals and Institutes; CCO, Curative Care Organization; OPD, Outpatient Department. *Source*: Mathauer et al.^4^

The pre‐Law health financing system is fragmented with several purchasing actors and different coverage schemes for different population groups, providing unequal benefit packages and using various payment methods, as depicted in Figure 1. For example, as one of the purchasing actors, the Health Insurance Organization, which is the main public insurer for 59% of the population, consists of several separate fund pools for the different population groups covered, for example, civil servants, retired civil servants, widowers of insured, pre‐school and school children and female‐headed households. Cross subsidies across these separate pools are restricted and subject to the Ministry of Finance approval. Another explicit health coverage scheme is the ‘Program for the Treatment at the Expense of the State’ (PTES) for the poor that are not covered by the Health Insurance Organization. This fragmentation limits the system's redistributive capacity with different levels of financial protection, and reduces efficiency due to high administrative costs.

In the public sector, purchaser‐provider integration has been the dominant arrangement. Public funds flow through a rigid line‐item budget, which is based, in most cases, on historical trends. Fee‐for‐service is the prevailing payment method by purchasers in the private sector, with no budget cap in place.

The nature of the healthcare provision market is even more fragmented: providers have varying levels of financial autonomy, receive multiple funding streams with different payment methods and contractual modalities, and provide diverse, loosely defined benefit packages.

The UHI Law introduces a purchaser‐provider split (Article 2 UHI Law). In this regard, three autonomous organizations have been created in mid‐2019: 1) the Universal Health Insurance Authority (UHIA) pools funds and purchases health services on behalf of the insured population; 2) the Healthcare Organization is in charge of healthcare service provision; and 3) the General Authority for Healthcare Accreditation and Regulation accredits healthcare providers (Articles 4, 15, 26 UHI Law). All public and private facilities must fulfil the required accreditation criteria after a transition period in order to be contracted by the UHIA (Article 31 UHI Bylaw).

**FIGURE 2:**
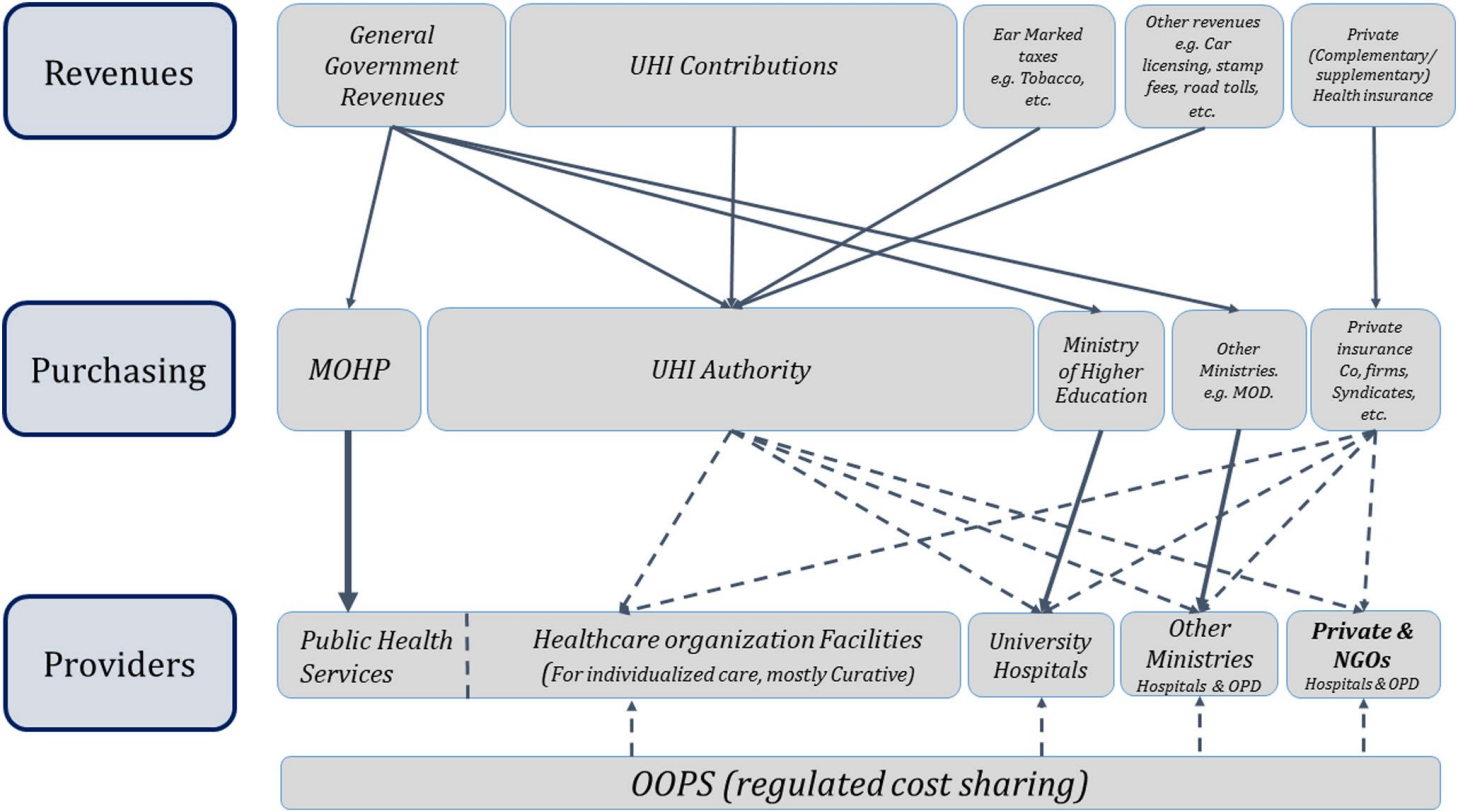
New health financing system architecture and funding flows as per the new UHI Law. Solid lines indicate line‐item payment method. Dotted lines, except for OOPS, indicate a contractual arrangement using most likely output‐oriented payment methods. UHI, Universal Health Insurance; MOHP, Ministry of Health and Population; MOD, Ministry of Defence; OPD, Outpatient Department. Mathauer et al.^4^

The Law merges the existing health financing schemes into one single pool for UHI (see Figure 2) allowing for increased financial risk spreading, purchasing power and efficiency. All Egyptians will be covered in this pool on a mandatory basis through family membership, except for staff and families of the military.

## 4 Implications of the UHI law: key shifts towards strategic purchasing and remaining challenges

This section presents an analysis of the institutional changes on purchasing arrangements, namely the design of benefits, the conditions of access (cost‐sharing mechanisms) as well as provider payment mechanisms (the process of setting them and their alignment). It looks into significant modifications made by the law in addition to challenges that may arise during the implementation.

### 4.1 Broad entitlements, but need for further specification

As per the UHI Law, the new benefit package is broad and generous since it contains ‘all diseases for diagnostic, therapeutic or rehabilitative purposes…’ (Article 3 UHI law). Experience from other countries suggests that if a benefit package remains broad and unspecified, implicit rationing (e.g., waiting lists, shortages) may arise or continue to prevail.^11^ There are several annotations in the Law, however, that refer to a list or package of services (Articles 3, 9 UHI Law and 3, 12 UHI Bylaw), suggesting that there are potential legal entry points for turning the broad benefit package into a more concrete list of defined services. Yet, the process for defining and revising the benefit package remains unclear in the legal provisions. For the pilot implementation phase, the UHIA opted to use the current Health Insurance Organization package as its basis for benefits specification.

Remarkably, the UHI benefit package does not cover prevention and does not mention explicitly early detection of diseases. The new health system architecture may lead to an even more explicit separation of curative services (financed through the UHI system) versus preventive and promotive services (financed by the Ministry of Health and Population), which may not help promote a focus on integrated people‐centered health services geared towards care coordination and continuity.

### 4.2 Cost‐sharing mechanisms aim at improved financial protection, yet these require further specification

The Law does not require cost‐sharing at the primary health care level or for consultations at higher levels of care except for medications, diagnostics and relatively low copayments for the inpatient departments (See Table 5). Furthermore, it puts ceiling on cost‐sharing and exempts the poor and vulnerable population and those with chronic conditions and tumours therefrom. This is particularly relevant to minimize the negative impact of cost‐sharing on access to health services and ensure financial protection of these population groups.

**TABLE 5:** Cost‐sharing mechanism as per the UHI Law

Medical services	Cost‐sharing rates and ceilings^a^
Home visit	100 EGP
Medications (except for chronic diseases and tumours)	10% up to a ceiling of 1000 EGP
	The percentage rises to (15%) in the tenth year of implementation of the Law
Radiology and all types of medical imaging (not related to chronic diseases and tumours)	10% of the total value up to a ceiling of 750 EGP per case
Medical and laboratory tests (not related to chronic diseases and tumours)	10% of the total value up to a ceiling of 750 EGP per case
Inpatient departments (except chronic diseases and tumours)	5% for a ceiling of 300 EGP per admission

^a^ 1 USD = 15.5 EGP (Egyptian pound). February 2020.

However, the period for ceiling amounts is not specified. For medications, it is not clear whether the ceiling amount refers to a period of one year or less or per one encounter. Neither is the list of copayment‐exempted chronic conditions with its related list of medications specified. This may lead to inconsistencies across providers at the point of service. Deliberations are taking place to preliminary enlist chronic conditions to be exempted.

### 4.3 Unclear process to determine and revise payment methods and rates

The Law and the Bylaw do not stipulate the process for setting payment methods for UHI covered services. Nonetheless the Law specified different arrangements for levels of care. For example, it foresees that beneficiaries register with a family healthcare unit or centre or family physician. This may suggest an implicit leaning towards some form of blended capitation payment for the primary level.

As per the UHI Law, a standing committee is established within the UHIA for setting payment rates for the list of covered health services, in coordination with the Healthcare Organization's oversight board (Article 9, 18 UHI Law). The committee includes representatives of public and private sector health service providers. This suggests that there will be a coordination/negotiation process within the committee. While the Law specifies the process of setting payment rates, this is not the case with respect to defining and reviewing the actual payment method. In fact, there is no multi‐stakeholder body (other than the Board of the UHIA) foreseen by the Law for this critical aspect.

### 4.4 Mixed provider payment system increasing incoherent incentives for providers

Personal preventive and promotive health services will be financed by the Ministry of Health and Population. These will continue to be governed by the current Budget Law and public financial management rules, which prescribe an input‐oriented line‐item based budget. As curative health services will be paid through output‐based payment methods by the UHIA, whereas preventive and promotive health services will be funded through line‐item budgets, health facilities (and staff) may find the payment characteristics of the UHIA more attractive. There is thus a risk that such a non‐aligned mixed payment system may lead to undesirable provider behaviour, namely resource shifting to curative care provision (such as shifting of staff time and attention, medical supplies, etc.), thus possibly resulting in resources shortages for preventive and promotive health care (cf. 3).

Because of the relatively extended implementation period, tertiary/university hospitals serving regions at different stages of implementation may receive different payment methods and rates for the different population groups. Such non‐aligned payments might possibly influence providers to treat more profitable patients (cream skimming) and engage in resource shifting for this patient group (cf. 3).

A summary of the main changes introduced by the UHI Law and the remaining problems are provided in Table 6.

**TABLE 6:** Summary of main changes introduced and remaining key challenges

	What did the law change and how does this contribute to more strategic purchasing?	What are the remaining challenges?
Benefits	‐ The UHI law implicitly refers to a list or package of services in an attempt to turn the broad list of benefits into an explicit package of services. ‐ Meanwhile, the UHIA is using an updated version of the current health insurance organization package as its basis for benefits specification.	‐ Undeveloped process and unclear criteria for setting priorities to include health services in the benefits package. ‐ Prevention and early detection are covered by the Ministry of Health and Population, not under the UHI package
Cost‐sharing mechanisms	‐ Cost‐sharing rates have been lowered, with a ceiling for medications, diagnostics and inpatient services. ‐ Poor and people with chronic conditions are exempted.	‐ For medications, the period for the ceiling amount is unclear. ‐ There is no specification for the list of chronic conditions with its related medications.
Process of setting provider payment	‐ The UHI provider payment methods have been separated from the government input‐oriented line item budget.	‐ The law does not specify the process of setting and revising the provider payment methods.
Alignment of provider payment methods	‐ UHI provider payments for curative services, though not stipulated by law, are paid through output‐based payments.	‐ The non‐aligned, mixed payment system (especially between preventive and curative services) may lead to undesirable provider behaviour, e.g., resource shifting.

## 5 POLICY OPTIONS AND CONCLUSIONS

This section summarizes the core options proposed to address the above concerns or challenges. These were discussed with key policy makers. Meanwhile, some of these options are being implemented.

### 5.1 Clarifying benefits

To specify and clarify the benefit package, it is suggested to establish a benefit package committee that will be in charge of taking decisions on defining, operationalizing and reviewing the benefit package on a regular basis. This should also involve the establishment of a clear process and criteria that reflect the policy priorities for the benefit package. Indeed, in 2019, a committee has been established within the UHIA to compile and explicitly enlist services across different levels of care based on the existing health coverage schemes and programs. Using evidence from a rigorous Health Technology Assessment will be critical, and it is proposed to expand and institutionalize the current Health Technology Assessment work in Egypt and to organize it independently from purchasers, providers or pharmaceutical actors. Dialogue around benefits design should also involve citizen and/or patient representatives so that the final decision taken by this committee represents the views of various population groups. These measures serve to not only increase transparency, but also enhance efficient use of scarce resources for health priorities and needs.

Finally, to ensure that payment incentives for providers are aligned (see next section), it is suggested to distinguish the benefit package into personal versus population‐based health services. As such, personal services, including promotive, preventive or curative care would also be covered by UHI and paid for by the UHIA. This ensures more efficient use of resources and better operationalization of benefits at the provider level, but would require an amendment to the UHI Law and/or Bylaw.

### 5.2 Revising and enforcing cost‐sharing rules to enhance equity

Cost‐sharing mechanisms could be further refined to be used as a policy tool to influence health seeking behaviour. To promote efficient allocation of resources, cost‐sharing could be reduced for generic drugs. Furthermore, lower or no cost‐sharing rates could apply to priority services, such as early detection and screening. In order to enhance equity in financing as well as financial protection, cost‐sharing rates could be differentiated between population groups, for example lower or no copayments for children, to enhance equitable access and financial protection. Finally, it is crucial to monitor the prohibition of balance billing. This also requires wide community‐based awareness on the entitlements as well as rules and referral regulation.

### 5.3 Clarifying the process of determining the payment methods and rates

It is proposed to explicitly specify the procedures for determining and reviewing the payment methods and rates, whether by an independent multi‐stakeholder commission or in coordination or negotiation between the UHIA, the Healthcare Organization and providers. In fact, a committee for establishing the provider payment methods was commissioned upon a decree by the Ministry of Health and Population in December 2018, and a temporary committee for strategic purchasing housed within the UHIA followed up on its work. This committee worked together with the pricing committee on different aspects of the payment system including adjustment of payment methods and rates, contracting modalities, medicine prescribing, etc.

### 5.4 Setting up an aligned mixed payment system with coherent incentives for providers

As the process of exploring and determining the final mix of payment methods is decided, it is important to assess and monitor the actual set of incentives created across the levels and types of care and to anticipate potential undesirable effects on provider behaviour. This will contribute to efficient service provision, quality of care and to equitable access of different population groups.

It is recommended to align the funding streams and provider payment methods for preventive and promotive care (government budget funded) with those for curative care (UHI payment methods) in order to avoid distortions in provider behaviour. If moving away from a budgeting approach based on line‐items for preventive and promotive care is not feasible within the short‐term, it is proposed to add a pay‐for‐performance component to give incentives to health workers to put more emphasis on such services.

*For outpatient care*, it has been suggested to apply a blend of partial capitation and fee‐for‐service for priority interventions plus performance payments in order to offset the undesirable incentives of each of these payment methods in isolation.^3^, ^12^ In May 2019, the UHIA board followed and approved the above recommended mix of payment methods for primary health care (PHC) services in the UHI pilot region. It also plans to incrementally include risk adjustment factors in the calculation of capitation rates such as age, sex and other socio‐economic characteristics, over the next few years as more detailed data become available, as was further recommended. International experience suggests that risk adjustment serves to reduce the incentive of under‐provision under capitation payments.^12^, ^13^, ^14^ Capitation as a payment method can also work for a family doctor operating in the private sector. To enhance quality of PHC, it will be useful to allow for competition between PHC facilities through patient choice during periods of open enrollments (e.g., 6 months or one year for example). For other specialized outpatient services, the UHIA decided to consider fee‐for‐service.

*For inpatient care,* the UHIA approved a mix of case‐based payment (for surgical interventions) and fee‐for‐service (for non‐surgical interventions) with a soft budget cap for the first phase of implementation. It has been decided to start with a simple system in terms of the number of cases and the rate setting. It is recommended that cases with frequent outlier costs should be treated differently. As the payment system matures, it can be incrementally refined to adjust for severity, comorbidities, etc. For equity concerns and to avoid the creation of a two‐tiered system, the (case) payment rate for the clinical intervention should be the same for all hospitals. Other systematic cost and price differences can be accounted for through adjustment factors (e.g., for University hospitals, or remote hospitals). With respect to fee‐for‐service payments, it is recommended to put a volume or budget cap on it to aim for budget neutrality and to limit expenditure growth.

In conclusion, the UHI law implementation implies major transformations in the health system that are assumed to bring about significant improvements and accelerate progress towards UHC. This analysis reveals and suggests that the proposed options and legal specifications are needed to support a shift towards more strategic purchasing in order to ensure that the UHI can make an effective contribution towards UHC. There is also need for more clarity in the institutional roles and responsibilities.

The Egyptian case of enacting and early implementation of the UHI Law provides several lessons for other countries in the region and beyond. While a legally binding mandate is essential to establish health financing and purchasing reforms, alignment across different elements of the reform is equally critical to ensure that purchasing reforms have a positive impact in order to improve access to care and financial protection. As part of this, it is important to consider the political economy that lies behind ‘technical’ options such as payment methods and rates or benefits specification. The political and institutional feasibility challenges related to these transformations need to be understood and addressed, as suggested by Mathauer et al.^1^ Moreover, effective governance arrangements are critical to support the move towards strategic purchasing.^15^ Likewise, strategic purchasing reforms must be aligned with other health systems and health financing policies. Finally, a clear implementation plan of the Law and its strategic purchasing aspects will be important to provide detailed orientation for the various actors.

## CONFLICT OF INTEREST

The authors declare that they have no competing interests.

## AUTHOR CONTRIBUTIONS

Inke Mathauer and Ahmed Khalifa conceived the original technical report with input from Awad Mataria.

Inke Mathauer and Ahmed Khalifa drafted the manuscript.

Jean Jabbour, Magdy Bakr, Awad Mataria and Mai Farid reviewed and commented on the draft manuscript.

All authors have read and approved the final manuscript.

## ETHICS STATEMENT

Not applicable.

## CONSENT FOR PUBLICATION

Not applicable.

## Data Availability

Part of the analysis is based on a review of the UHI Law and Bye Law (see references Government of Eygpt^5^, ^6^). It is published in the Egypt Gazette, available here: https://www.youm7.com/story/2017/12/18/%D9%86%D9%86%D8%B4%D8%B1‐%D9%86%D8%B5‐%D9%82%D8%A7%D9%86%D9%88%D9%86‐%D8%A7%D9%84%D8%AA%D8%A3%D9%85%D9%8A%D9%86‐%D8%A7%D9%84%D8%B5%D8%AD%D9%89‐%D8%A7%D9%84%D8%B4%D8%A7%D9%85%D9%84‐%D8%A8%D8%B9%D8%AF‐%D9%85%D9%88%D8%A7%D9%81%D9%82%D8%A9‐%D8%A7%D9%84%D8%A8%D8%B1%D9%84%D9%85%D8%A7%D9%86‐%D8%A7%D9%84%D8%AF%D9%88%D9%84%D8%A9/3561419. (An English translation of the Law and Bye‐Law was used for the analysis).
